# A Case Report on Familial White Sponge Nevus in Saudi Arabia

**DOI:** 10.7759/cureus.32674

**Published:** 2022-12-18

**Authors:** Sara Akeel, Sarah AlFarabi, Sara Binsaad, Soulafa Almazroa, Nada Binmadi

**Affiliations:** 1 Oral and Maxillofacial Pathology, King Abdulaziz University, Jeddah, SAU; 2 Oral and Maxillofacial Pathology, King Abdulaziz University Hospital, Jeddah, SAU; 3 Dentistry, King Abdulaziz University, Jeddah, SAU

**Keywords:** gene mutations, oral mucosal lesions, hyperkeratosis, genodermatosis, white sponge nevus

## Abstract

White sponge nevus (WSN) is an uncommon, benign, autosomal dominant disorder that usually appears at birth or in early childhood. It affects males and females equally and is caused by germ line mutations of the keratin genes leading to keratin instability and tonofilament aggregation. The condition causes painless, white, thickened, corrugated plaques to form on the oral mucosa, especially bilaterally on the buccal mucosa. Extra-orally, it occurs most often in the vaginal mucosa, as well as in the nasal and esophageal mucosa. In this report, we describe the case of a healthy 32-year-old Saudi male in Jizan in southern Saudi Arabia whose general dentist referred him to the oral medicine clinic at King Abdulaziz University Hospital, where he was diagnosed with white sponge nevus. The patient reported no medical problems and was a cigarette smoker for more than 10 years. An oral examination revealed white lesions affecting the buccal mucosa bilaterally and the labial mucosa. A biopsy of the buccal mucosa confirmed the diagnosis of white sponge nevus. Laser therapy was suggested for the aesthetic treatment of the lesions. Better awareness of this hereditary condition among dental professionals can help improve timely diagnoses early in life and thus avoid unnecessary or inadequate treatment for this benign condition.

## Introduction

White sponge nevus (WSN) is a rare, benign, and autosomal dominant disorder caused by germ line variants of the keratin genes, specifically *cytokeratin 4* (*KRT4*) and *cytokeratin 13* (*KRT13*), on chromosomes 12q13 and 17q21-q22, respectively [1]. The first report was documented by Hyde in 1909, and the condition was further described by Cannon in 1935 as “white sponge nevus” [1]. Typical onset is early in life, and it occurs at similar rates in males and females [2]. Clinically, WSN appears as painless white or gray plaques that are diffused, thickened, and corrugated or velvety and do not disappear with the stretching of the tissue. It mostly occurs in the oral mucosa on the bilateral buccal mucosa, lips, alveolar ridges, and floor of the mouth [3]. Extra-oral sites have been shown to arise only from *KRT13* variants [1], most commonly in the vaginal mucosa, as well as in the laryngeal and esophageal mucosa. A recent publication by Joseph et al. reports that around 30% of biopsied lesions are mucosal pathologies, with hyperkeratosis accounting for most [4]. However, little is known about the incidence or prevalence of WSN in the Arab world. We report a case of WSN in an adult patient who was diagnosed after multiple visits to numerous dentists due to the lack of awareness about the condition.

## Case presentation

**Figure 1:**
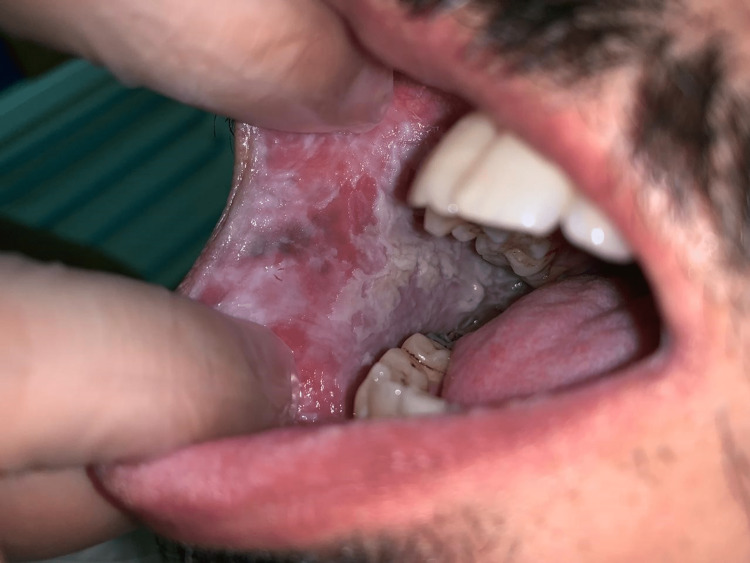
Clinical picture of the right buccal mucosa White plaque with irregular well-defined borders on the right buccal mucosa

**Figure 2:**
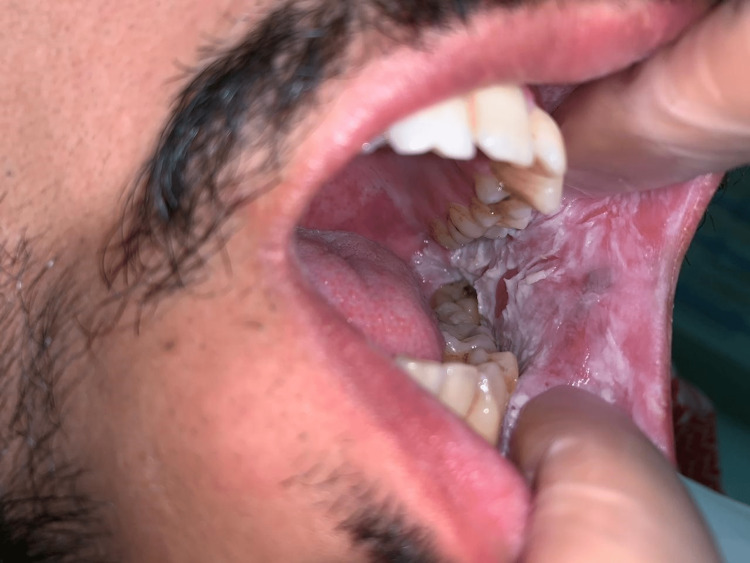
Clinical picture of the left buccal mucosa White plaque with irregular well-defined borders on the left buccal mucosa

**Figure 3:**
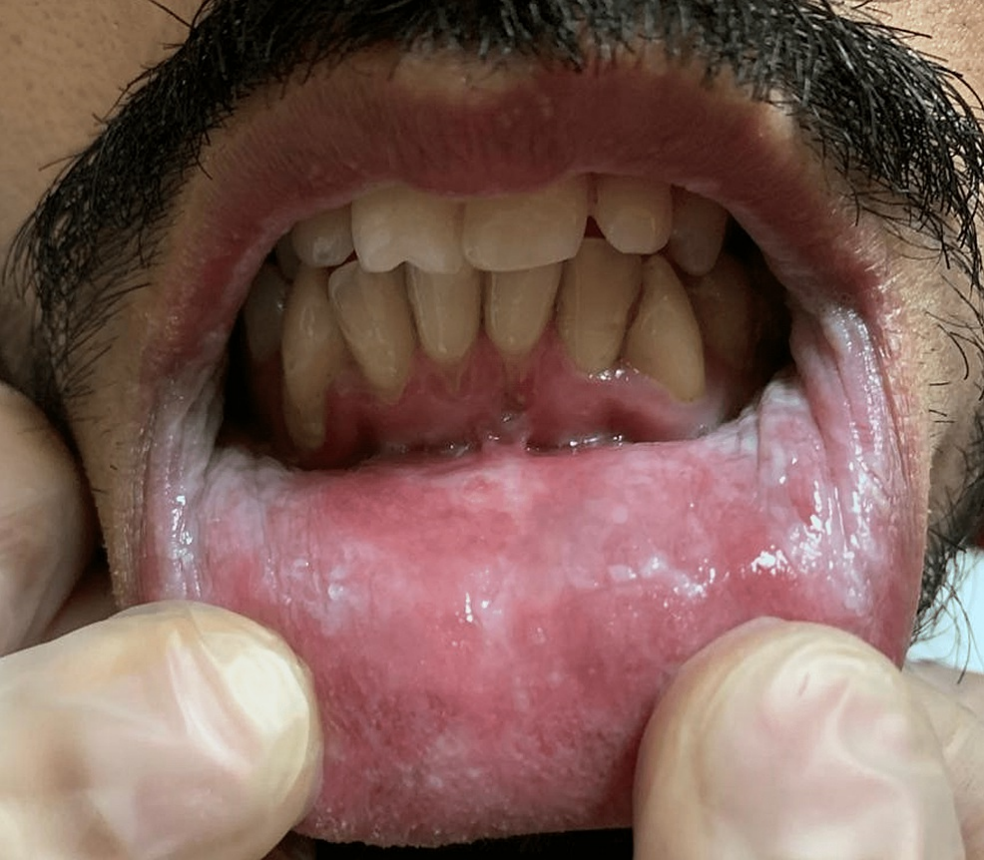
Clinical picture of the lower labial mucosa White plaques of the lower labial mucosa

**Figure 4:**
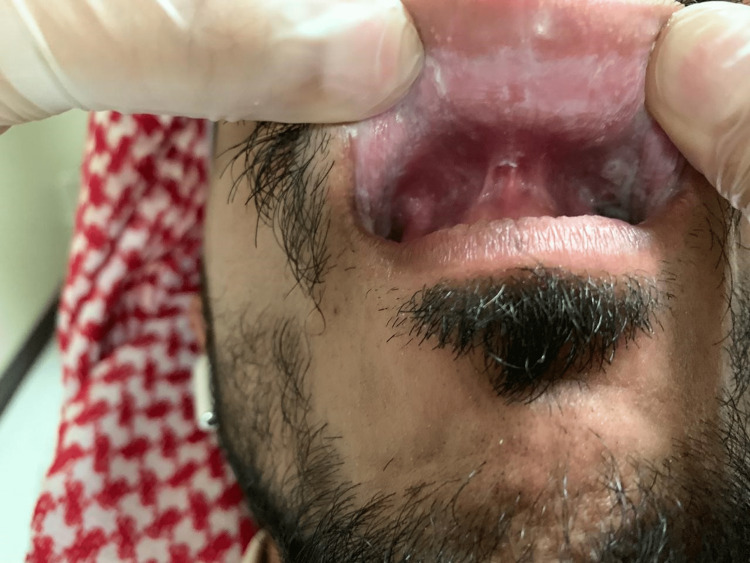
Clinical picture of the upper labial mucosa White plaques of the upper labial mucosa

A 32-year-old Saudi male was referred to the oral medicine clinic at King Abdulaziz University Hospital in Saudi Arabia for periodontal issues. The patient also complained about the appearance of his mouth. The extra-oral examination was within normal limits. The intraoral examination revealed diffuse ragged grayish-white plaques bilaterally on the buccal and labial mucosa (Figures 1-4). The patient was aware of the lesions and reported biting them frequently.

**Figure 5:**
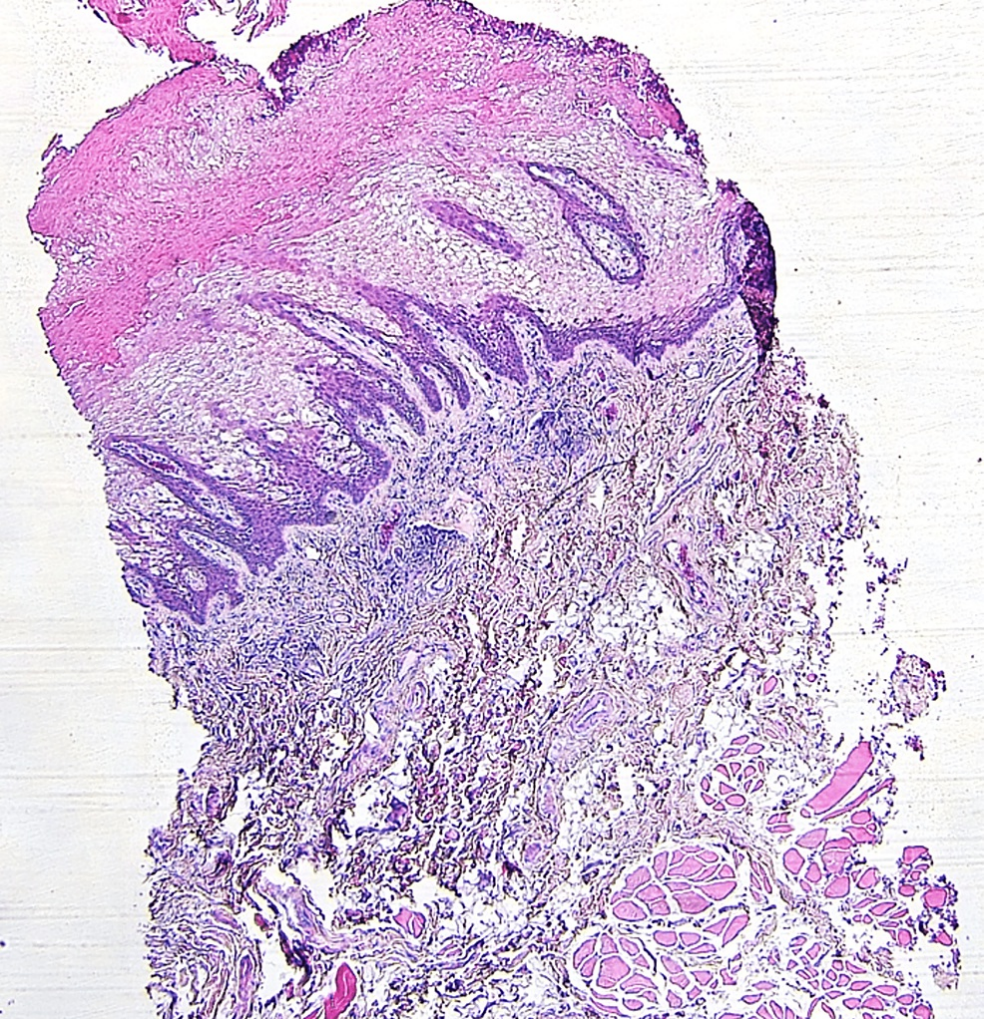
Histopathological features indicating white sponge nevus (low power) The epithelium exhibits hyperparakeratosis and acanthosis with a pale “spongy” appearance

**Figure 6:**
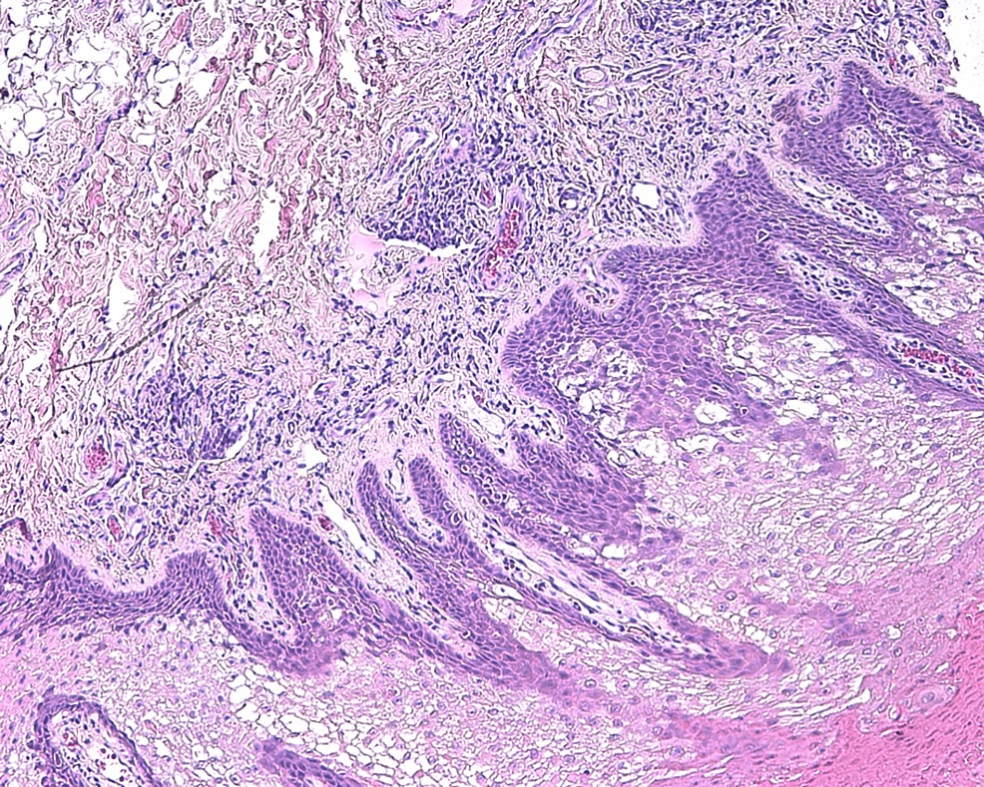
Histopathological features indicating white sponge nevus The pale epithelium is caused by intracellular vacuolation and dyskeratosis that spares the basal cells

**Figure 7:**
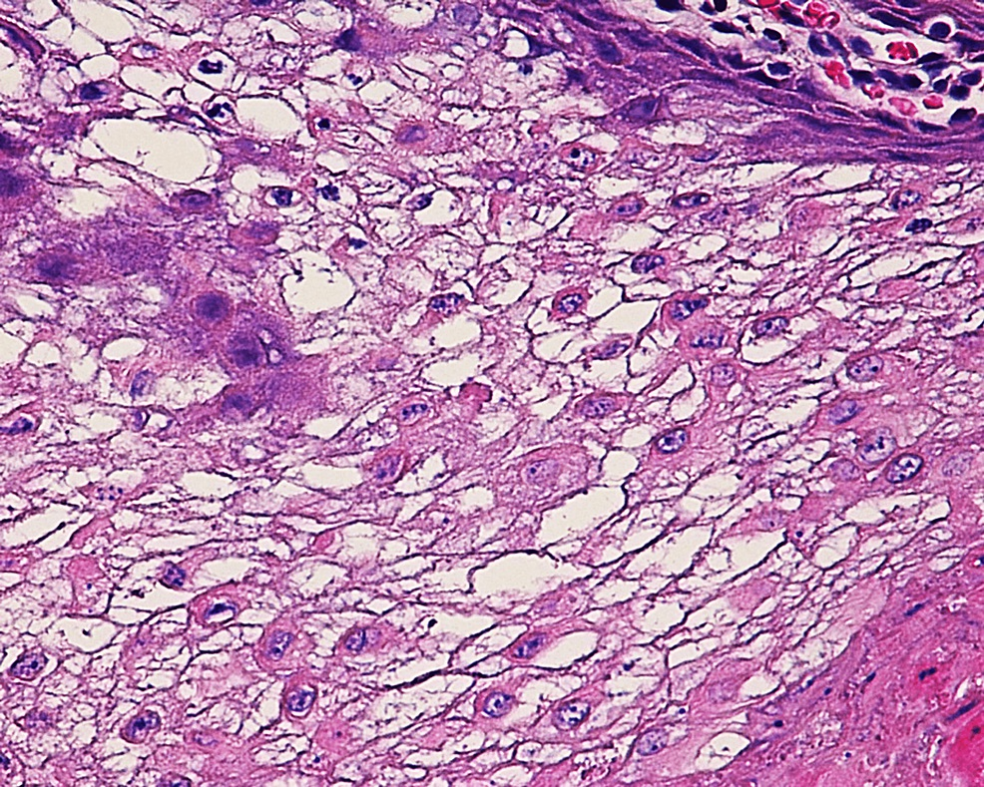
Histopathological features indicating white sponge nevus (high power) Perinuclear eosinophilic condensations and intracytoplasmic vacuolation (not spongiosis)

A biopsy of the left premolar-molar buccal mucosa was submitted for microscopical evaluation and revealed parakeratosis, acanthosis, and spongiosis at the epithelial surface (Figure 5). Extensive vacuolation of suprabasal keratinocytes was observed, as well as dyskeratotic cells exhibiting dense perinuclear eosinophilic condensation (Figures 6-7). There was no evidence of dysplasia or candidal hyphae.

Genetic testing was not conducted due to a lack of availability. Based on the patient’s histopathological results and clinical features, a diagnosis of WSN was achieved. The patient was asked to bring his father and brother to the clinic to be examined, but they refused. Laser management was suggested to improve the aesthetics, which was the main complaint of the patient, but he did not return for further treatment.

## Discussion

WSN is a form of genodermatosis inherited as an autosomal dominant disorder. It usually appears early in life with no gender preference. In the current case, the lesions were diagnosed at the age of 32 when the patient was referred to King Abdulaziz University Hospital for a periodontal problem. He had been seen by numerous dentists complaining of the appearance of his cheek but never received a proper diagnosis, likely because the condition is not well-known, has a low incidence rate, and resembles other common conditions of the oral cavity such as leukoedema and candidiasis.

Keratins are a group of about 30 proteins expressed in the cytoplasm of epithelial cells to form a dense network of intermediate filaments or scaffolding. The keratinocytes in this scaffolding presumably provide resistance to trauma. Mutations in the genes that encode these proteins result in a variety of diseases characterized by epithelial fragility and hypertrophy [5], including WSN.

The literature reports 83 reported cases of WSN worldwide, with only a few in the Arab world. For example, a six-year-old female in Casablanca, Morocco, presented to the dermatology department with chronic white lesions of the oral mucosa. No other family member reported similar symptoms, and no treatment was performed due to the benign and asymptomatic nature of the lesions [6]. A study conducted on tongue diseases in a Libyan population between July 2007 and February 2008 identified three patients with WSN exhibiting irregular epithelial surface keratosis on the lateral aspect of the tongue [7]. Kuwait University also conducted a study to determine the range, frequency, prevalence, and distribution of oral lesions. Although the results did not identify an exact number of WSN cases, the most common diagnostic category was mucosal pathologies, mainly histopathological diagnoses such as hyperkeratosis, followed by odontogenic cysts and reactive lesions [4]. To our knowledge, WSN has not been reported in Saudi Arabia mostly due to the rarity of the condition.

The diagnosis of WSN in cases with no familial pattern is challenging. Differential diagnosis may include oral leukoplakia, leukoedema, chronic cheek biting, hyperplastic candidiasis, and the plaque variant of lichen planus. Even in the presence of distinctive characteristics of hereditary WSN, other conditions such as congenital pachyonychia, congenital dyskeratosis, Darier’s disease, benign inherited acanthosis nigricans, and hereditary benign intraepithelial dyskeratosis must be excluded [8]. WSN is commonly misdiagnosed as candidiasis, oral leukoplakia, or squamous cell carcinoma. Consequently, achieving an accurate diagnosis is paramount to avoid unwarranted worry or improper management [9].

Although WSN is mostly asymptomatic, causing a benign increase in the growth of epithelial cells, the excess tissue can promote bacterial or fungal growth leading to infection and pain. Furthermore, changes in physical appearance, especially in the oral mucosa, can be bothersome [10], leading to chronic cheek biting, as in the current patient. Histopathological features include epithelial thickening with hyperparakeratosis and spongiosis, extensive vacuolization of the supra-basal keratinocytes, the absence of subepithelial inflammation, and a characteristic but not necessarily pathognomonic perinuclear eosinophilic condensation corresponding to tonofilaments aggregates [6]. None of these histopathological attributes are specific to WSN, but the presence of one or more can aid in the diagnosis [5].

Interestingly, an association between WSN and ectrodactyly-ectodermal dysplasia-clefting (EEC) syndrome has been reported. This rare disorder is characterized by abnormalities of the hands, feet, skin, hair, and teeth, including cleft lip and palate [11]. Considering the low prevalence rates of both disorders [12], co-existence could be coincidental. However, three cases have reported an association between WSN and EEC [13,14]. Thus, EEC syndrome presenting with ectrodactyly and with intraoral white plaques that are confirmed by histopathology to be WSN could be linked to a genetic alteration [14]. Future studies should investigate molecular biology techniques to identify relevant interactions between chromosomal regions 7q11.2-q21.3, which are involved in EEC syndrome, and 12q13 and 17q21, which are related to mucosa-specific keratins [12].

WSN does not usually require management except patient education and reassurance. No standard protocol has been established, but treatment often includes improving the mucosal aesthetics and texture [15]. Partial remissions have been recorded with the use of tetracycline mouth rinses and long-term, low-dose systemic antibiotic therapy [16]. Antibiotic or antifungal mouth rinses can treat any superimposing infection, if present, but do not affect the course of the disease. A better understanding of the disease mechanism is needed to develop effective therapies [9].

In the current case, the patient was concerned by the appearance of the mucosa. Accordingly, laser therapy was suggested, although current evidence does suggest effective results [16]. As mentioned, the patient was a tobacco smoker, which increases the risk of developing potentially malignant conditions and oral cancer. A biopsy was performed to confirm the diagnosis and rule out any dysplastic change. However, symptoms of WSN include extensive involvement of the oral mucosa, which could make it difficult for the patient to notice any new or alarming changes. The patient was educated and advised to continue with regular follow-up and to consider smoking cessation.

## Conclusions

To our knowledge, this is the first case report on WSN in Saudi Arabia. Further awareness of this hereditary, benign condition can help dentists identify this condition at an earlier age, thus avoiding improper management and inadequate treatments, as well as reassuring young patients and their parents about the benign character of the lesions. Patient education and follow-up are crucial, especially among patients at risk of oral cancer due to risk factors such as smoking. In addition, epidemiological studies across the Arab world are needed to assess the prevalence and incidence of this condition and further promote awareness among dental professionals.
